# Evaluation of the Antibacterial and Antifungal Properties of* Phragmanthera capitata* (Sprengel) Balle (Loranthaceae), a Mistletoe Growing on Rubber Tree, Using the Dilution Techniques

**DOI:** 10.1155/2017/9658598

**Published:** 2017-05-31

**Authors:** Franklin Uangbaoje Ohikhena, Olubunmi Abosede Wintola, Anthony Jide Afolayan

**Affiliations:** Medicinal Plants and Economic Development (MPED) Research Centre, Botany Department, University of Fort Hare, Alice, South Africa

## Abstract

The alarming increase in multidrug resistance of pathogenic microorganisms to conventional drugs in recent years has prompted the search for new leads in alternative remedies in natural products. Hence, this study was aimed at evaluating the antimicrobial properties of* Phragmanthera capitata*, a parasitic mistletoe growing on rubber trees. The in vitro antimicrobial activities of the acetone, methanol, ethanol, and aqueous extracts were investigated using five gram-negative and five gram-positive bacteria and four fungi. A 96-well resazurin broth and agar dilution techniques were used for the determination of the Minimum Inhibitory and Bactericidal Concentrations. The antibacterial activity of the organic extracts had comparative effects on all the bacteria with a MIC of 1.25 to 5 mg/mL and MBC of 2.5 to 10 mg/mL. However, the acetone extract showed higher bactericidal effect while the aqueous extract was not active. The organic solvent extracts also showed antifungal activities on two of the fungi with a MIC of 1.25 mg/mL to 10 mg/mL. However, the aqueous extract had the highest activity inhibiting all the fungi with a MIC of ≤0.3125 to 1.25 mg/mL. The study supports the ethnomedicinal claims of* P. capitata* as a remedy for the diseases/infections caused by these organisms.

## 1. Introduction

The advent of antibiotics has proved to be the main tool in combating microbial infections and has greatly improved the health-related qualities of human life. Since the discovery of antibiotics and their uses in chemotherapy, there has been a notion in the health sector that this would lead to the eventual eradication of infectious diseases. However, over the past decades, overreliance and use of antibiotics have led to the emergence and dissemination of multidrug resistant strains of several groups of microorganisms [1, 2].

Due to the increase in resistant clinical isolates, there is a paramount need to develop new and innovative antimicrobial agents [3]. Coates et al. [4], cited by Khan et al. [1], stated that even new families of antimicrobial agents will result in short life expectancy because of reports on the rapid and widespread emergence of resistance to newly introduced antibiotics. Therefore, researchers are looking for new leads in the discovery of better alternatives against multidrug resistant microbial strains. Among the potential sources of new agents, plants have long been investigated owing to their popular use as remedies for diverse infectious diseases because they contain many bioactive compounds that could be of interest in therapeutics [3]. Herbal drugs research is increasing on a daily basis not only because they serve as lead to the formulation of new preventive or curative drugs, but also because they are affordable and believed to be safer than orthodox drugs [5–7].

One of the groups of plants popular for its folkloric usage and for the treatments of all kinds of ailments is the mistletoe also commonly known as “heal all” because of the belief that it can cure all diseases [8]. Among the three known families of the mistletoe, Loranthaceae is widely distributed and extensively exploited in Africa for its diverse therapeutic values. Some of the recognised therapeutic applications include antitumor, cough, headache, tightening of the uterus after childbirth, antiviral, anticancer, antinociception, and antimicrobial [9, 10].

*Phragmanthera capitata* (Sprengel) Balle, is a mistletoe of the Loranthaceae family. As with most mistletoe, it is a medicinal plant which has been used in Africa traditional medicine for the treatment of diverse diseases [11]. It is a parasite of different economic plants like cocoa, kola, coffee, mango, almond, rubber tree, and so on [5].

Prior to this study, there had been a dearth or no information on the antimicrobial activities of* Phragmanthera capitata* growing on rubber tree saving the report of Ogunmefun et al. [5] of* Phragmanthera incana* growing on kola nut and cocoa. This may be due to the fact that most studies on mistletoe are focused on the destructive habit and physiological interaction on host plants. Since the bioconstituents of mistletoes are chiefly dependent on their host plant, this work, therefore, aims to evaluate the antibacterial and fungal activities of* P. capitata* that is parasitic on rubber tree* (Hevea brasiliensis)* on different human and animal pathogenic strains of microorganisms using the agar and broth dilution techniques.

## 2. Materials and Methods

*Collection of Sample.* The leaves of* P. capitata* were collected from mature rubber plantations in the Rubber Research Institute of Nigeria. The site is located on latitude 6°00′–6°15′N, on longitude 5°30′–5°45′E, and at about 27 m above sea level. The sample was authenticated by Dr. Emmanuel I. Aigbokhan of the Plant Biology and Biotechnology Department, University of Benin, Edo State, Nigeria. A voucher specimen (UBH10284) was deposited at the UNIBEN herbarium for future reference.

### 2.1. Extraction Procedure

Leaves were removed from the twigs, rinsed gently to remove dust and dirt, air-dried at room temperature (mean morning and night temperature of 24°C and mean noon temperature of 27°C) in a well-aerated atmosphere, and prevented from direct sunlight to avoid denaturation of vital phytoconstituents. Dried leaves were pulverised. One hundred grams each of the ground sample was extracted by maceration with acetone, methanol, ethanol, and water and shaken in an orbital shaker (Orbital Incubator Shaker, Gallenkamp) for 24 hours. The crude extracts were filtered using a Buchner funnel and Whatman number 1 filter paper. The acetone, methanol, and ethanol extracts were further concentrated to dryness to remove the solvents under reduced pressure using a rotary evaporator (Strike 202 Steroglass, Italy) while the aqueous filtrate obtained was concentrated using a freeze dryer (Vir Tis benchtop K, Vir Tis Co., Gardiner, NY).

### 2.2. Rationale for the Selection of the Microorganisms

The bacteria and fungi used for this work were selected based on their roles as opportunistic pathogens to humans and animals and their association with stomach disorders, diarrhoea, dysentery, wound, and other infections and primarily to validate the ethnopharmacological claims of* P. capitata* as a remedy for these diseases [5].

### 2.3. Microbial Strains

All the organisms used in this study were obtained from the Medicinal Plants and Economic Development (MPED) Research Centre, University of Fort Hare, South Africa. Five gram-positive strains,* Enterococcus faecalis* (ATCC 29212),* Staphylococcus aureus* (OK),* Bacillus subtilis* KZN,* Bacillus cereus, *and* Streptococcus pyogenes*, and 5 gram-negative strains,* Vibrio cholera, Klebsiella pneumonia* (ATCC 4352),* Pseudomonas aeruginosa* (ATCC 19582),* Salmonella typhi* (OK), and* Escherichia coli* (ATCC 8739), were used for the antibacterial activity. The fungi isolates used were* Trichophyton mucoides* ATCC 201382,* Trichophyton tonsurans* ATCC 28942,* Candida albicans* (ATCC 10231), and* Aspergillus niger* ATCC 16888.

### 2.4. Preparation of Bacterial Inoculum

Direct colony suspension method was used in preparing the inoculum. Three to five morphologically similar colonies from fresh Muller Hinton Agar plates were transferred with a loop into about 5 mL of normal saline in a capped test tube and vortex. The suspension formed was adjusted to give a turbidity equivalent to that of a 0.5 McFarland standard (BaSO_4_ prepared spectrophotometrically) to give an approximate 1.5 × 10^8^ CFU/mL. The adjusted colony was then diluted in a ratio 1 : 100 in Muller Hinton Broth to give a colony suspension of 1 × 10^6^ CFU/mL. Final suspensions of 1 × 10^4^ CFU/spot and 3–7 × 10^5^ CFU/mL were used for the agar and broth dilutions, respectively.

### 2.5. Preparation of Fungal Inoculum

Fungal strains were freshly subcultured on sterile Sabouraud Dextrose Agar and incubated at 30°C for 2–5 days. The resultant cells and spores were washed into sterile normal saline and the turbidity adjusted to a 0.5 McFarland standard equivalent. This results in a 1 × 10^6^ CFU/mL. The suspension is further diluted in a 1 : 10 ratio in Sabouraud Dextrose Broth to give a turbidity of 5 × 10^5^ CFU/mL.

### 2.6. Dilution Assays

Agar dilution and broth microdilution assays as described by Wiegand et al. [12] and the European Committee for Antimicrobial Susceptibility Testing (EUCAST) [13] which are modifications from the guidelines of the Clinical and Laboratory Standard Institute (NCLI) were used for this study.

### 2.7. Preparation of Extract

A stock solution of 500 mg/mL that was first dissolved in a little amount of DMSO and made up with either Muller Hinton or Sabouraud Dextrose Broth for antibacterial and antifungal assays, respectively, was prepared. Twofold serial dilutions of the stock (250, 125, 62.5, 31.23, 15.625, and 7.8125 mg/mL) were also prepared in broth. Standard drugs (ciprofloxacin and nystatin for antibacterial and fungi, resp.) were also prepared in 2-fold serial dilutions according to the guidelines of the Clinical and Laboratory Standard Institute.

### 2.8. Resazurin (Alamar Blue) Preparation

Resazurin was obtained as a tablet and prepared according to the manufacturer's specification. A tablet was dissolved in 50 mL of sterilised distilled water and vortex. A ratio 1 : 10 final volume was used for the assay.

### 2.9. Agar Dilution Assay

Muller Hinton and Sabouraud Dextrose Agar were, respectively, prepared according to the manufacturer's description for antibacterial and fungi screening. The agar was autoclaved at 121°C for 15 min and allowed to cool to 50°C in a water bath. About 0.5 mL from the 2-fold serial dilutions was added to the molten agar (24.5 mL) in the water bath, swirled and poured into Petri dishes, and allowed to cool and solidify. Ten microliter (10 *μ*L) each from both the prepared bacterial and fungal inoculum was delivered on the solidified agar surface to give the desired final inoculum of 1 × 10^4^ CFU/spot and 1 × 10^3^ CFU/mL, respectively. The extract concentrations for the antibacterial ranged from 5 mg/mL to 0.1563 mg/mL while for the antifungal assay, a range of 10 mg/mL to 0.3125 mg/mL was used. The concentration of ciprofloxacin (antibacterial standard) ranged from 64 *μ*g/mL to 2 *μ*g/mL while nystatin (antifungal standard) ranged from 16 *μ*g/mL to 0.5 *μ*g/mL. Bacteria plates were incubated at 37°C and readings were taken between 16 and 20 hrs and after 3 days of incubation; fungi plates were incubated at 30°C and initial readings were taken after 2 to 3 days and the second reading was taken after 5 days.

### 2.10. Broth Microdilution Assay

Muller Hinton Broth for antibacterial screening was also tested using the 96-well microtiter plate with lid. The extracts and the standard drug were prepared in a concentration twice the desired final concentration as it will be diluted with an equal amount of bacteria in broth. Briefly, 200 *μ*L of the prepared extracts and standard drug in broth was introduced into the first wells in columns 1–10 (in row A). Rows B–H in columns 1–10 had 100 *μ*L of broth alone while rows A–H in column 11 had 200 *μ*L of broth and 100 *μ*L of broth was in A–H in column 12. Twofold serial dilutions using a multichanneled micropipette was done systematically down the columns 1–10 (from rows B–H). 100 *μ*L was removed from the starting concentrations (columns 1–10 in row A) and transferred to the next row with the 100 *μ*L broth, properly mixed, and the procedure was repeated up to the last row (H) where the last 100 *μ*L was discarded. This brings the final volume in all the test wells with the extracts and the standard drugs to 100 *μ*L except the 11th column which had 200 *μ*L of the broth that served as sterility control. An equal volume (100 *μ*L) of the 1 × 10^6^ CFU/mL bacterial inoculum was transferred into all the wells except the 11th column to give us the desired final inoculum load of 5 × 10^5^ CFU/mL. Column 12 served as growth control (drug-free). The extracts concentrations ranged from 10 mg/mL to 0.078 mg/mL while ciprofloxacin ranged from 2 *μ*g/mL to 0.0156 *μ*g/mL. Microtiter plates were incubated at 37°C for 18–20 hrs. After incubation, 20 *μ*L of Alamar blue (resazurin) was added to all the wells and incubated for few minutes to observe any colour changes.

### 2.11. Minimum Inhibitory Concentrations/Minimum Bactericidal Concentrations (MIC/MBC)

The Minimum Inhibitory Concentrations were determined visually in the agar and broth dilutions as the lowest concentrations of the extracts at which no bacterial/fungal growth was visible (or greatly reduced in comparison to the controlled growth in the antifungal assay) or colour changed from blue to pink in the case of the resazurin broth assay. Minimum Bactericidal Concentrations were determined by subculturing wells with no colour change on fresh agar plates and incubated at 37°C for 16 to 20 hrs. After the incubation, the lowest concentration that did not show any visible growth was taken as the MBC.

## 3. Results

**Figure 1:**
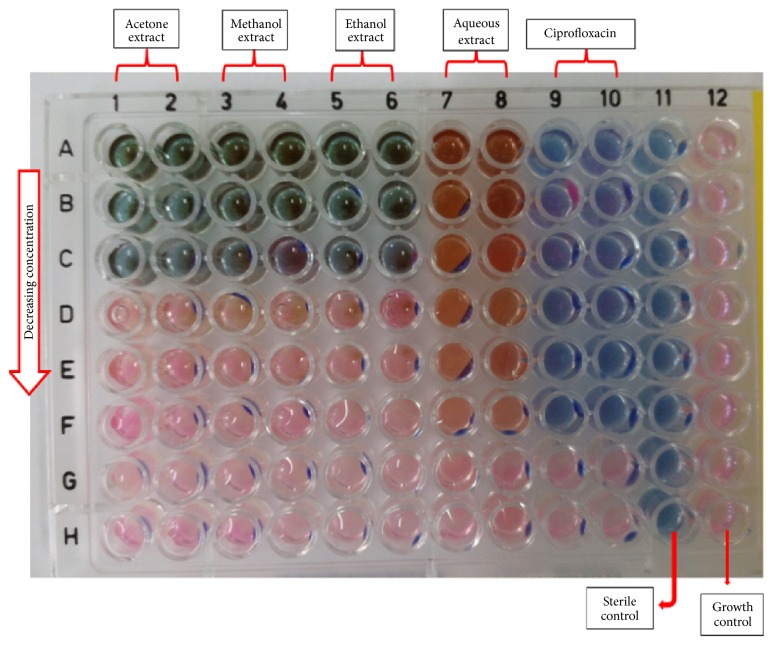
*A schematic representation of the 96-well resazurin broth microdilution model*. Annotations: The blue colouration indicates inhibition of growth; pink indicates that organisms are active. The different shades of blue and pink colours in columns 1–8 as compared to columns 9–12 are due to the different extracts colours.

The results of the antibacterial Minimum Inhibitory Concentration (MIC) using agar dilution and resazurin broth microdilution assays (Figure 1) are shown in Table 1. The result revealed that both gram-positive (+ve) and gram-negative (−ve) bacteria tested were susceptible to the crude extracts of* P. capitata. *The gram-negative bacteria were more susceptible to the crude extracts in both methods assayed for but more defined in the agar dilution. The MIC values in the agar dilution ranged from 1.25 mg/mL to 2.5 mg/mL for all the gram-negative bacteria except for* K. pneumonia* (in the methanol and ethanol extracts) and* S. typhi* (in the methanol extract) which had MIC values of 5 mg/mL. The aqueous extract had the lowest activity compared to the organic solvent extracts but also exhibited better gram-negative activity in the agar dilution method with a MIC value of 5 mg/mL in* E. coli, S. typhi, *and* V. cholera.* However, there was no activity recorded for the aqueous extract in the broth dilution technique. The standard drug (ciprofloxacin) showed great antibacterial activity with a MIC value ranging from 0.0625 *μ*g/mL to 0.25 *μ*g/mL in the broth dilution and ≤2 *μ*g/mL (lest concentration tested) in the agar dilution.

Table 2 is the result of the bactericidal activity of the extracts and agar dilution MIC after three days of incubation. The organic solvent extracts of* Phragmanthera capitata* showed more bactericidal activity on* Escherichia coli* with a MBC value of 2.5 mg/mL while there was no bactericidal activity on* P. aeruginosa.* The MBC for the organic extracts ranged from 2.5 mg/mL to 10 mg/mL with acetone having the best activity. The result of the agar dilution technique incubated for a prolong time (3 days) showed a comparable result to the MBC with a range of 2.5 mg/mL to values greater than the highest concentration tested (>5 mg/mL) for the crude extracts. Ciprofloxacin also showed a great lethal activity on almost all the organisms at the concentrations tested except for* E. faecalis* that survived the highest dosage for this test. However, variation was observed in the organisms incubated for a prolonged period of time with ciprofloxacin in the agar. While, at 4 mg/mL,* V. cholera* and* K. pneumonia *continued to grow, the same organisms were killed in the bactericidal test.

**Figure 2:**
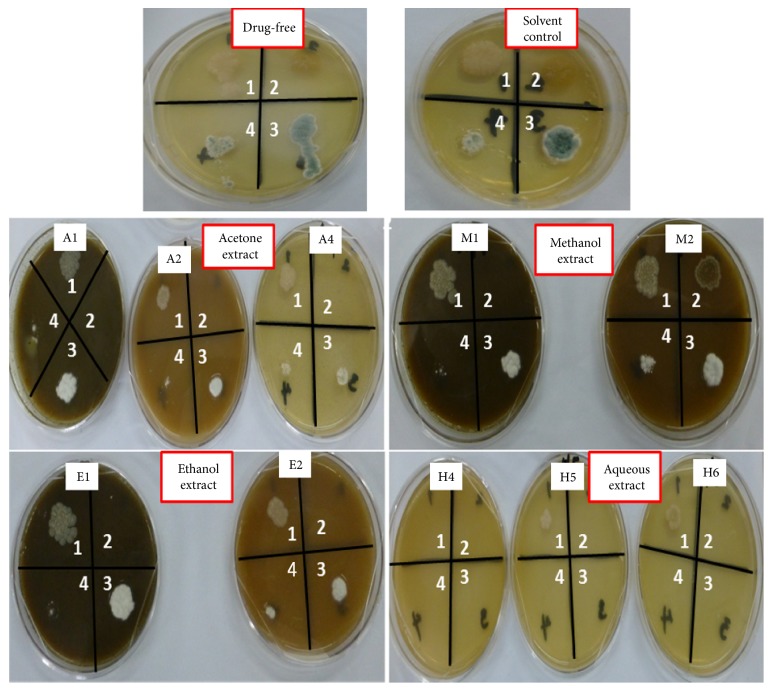
*Representation of the antifungal MIC determination of P. capitata against the tested fungi using the agar dilution technique*. Annotations: Values 1–4 are the fungi used which are as follows: 1 =* Trichophyton mucoides*; 2 =* Trichophyton tonsurans; *3 =* Candida albicans;* and 4 =* Aspergillus niger. *A1, A2, and A4, M1 and M2, E1 and E2, and H4, H5, and H6 are the different concentrations used with the highest concentration being 1 (10 mg/mL) and the least concentration being 6 (0.3125 mg/mL). Spots on plates are fungi growth indicating resistance of the organism(s) at that concentration.

The antifungal activities of the solvent extracts of* P. capitata* on some selected human pathogenic fungi are as shown in Table 3. The MIC were read twice: between 2 and 3 days and after 5 days of incubation. The MIC for* C. albicans *and* A. niger* were taken as the concentrations which showed little spot to no growth in comparison to the control (Figure 2). The result revealed that* T. mucoides* and* C. albicans* were resistant to the organic solvent extracts of the plant.* Trichophyton tonsurans* and* A. niger* were, however, susceptible to the organic solvent extracts. The MIC for acetone, methanol, and ethanol extracts on* T. tonsurans* were 1.25 mg/mL, 10 mg/mL, and 5 mg/mL, respectively. Acetone and ethanol extracts had MIC of 5 mg/mL while methanol extract had MIC value of 10 mg/mL on* A. niger*. The result after 5 days of incubation was similar to the first observation except in the ethanol extract where the MIC increased from 5 mg/mL to 10 mg/mL in both* T. tonsurans* and* A. niger*. However, a very high susceptibility was observed in the aqueous extract in all the fungi assayed and remained unchanged after 5 days of incubation. The MIC of the aqueous extract was 1.25 mg/mL on* T. mucoides* and ≤0.3125 mg/mL (lowest concentration tested) on* T. tonsurans, C. albicans, *and* A. niger. *Nystatin also showed a high MIC on the tested organisms with MIC which ranged from 4 *μ*g/mL to 8 *μ*g/mL after 2-3 days of incubation and was greater than 16 mg/mL in* C. albicans* after 5 days of incubation.

## 4. Discussion

In ethnopharmacological research, antimicrobial susceptibility tests are carried out to determine how effective potential antimicrobial agents from biological extracts could be against different pathogenic microorganisms. These tests are used to screen plant extracts for antimicrobial activities and also used to determine or ascertain the usefulness of antimicrobial agents in fighting infections by determining their MIC [14]. According to the EUCAST document [13], in vitro susceptibility tests are carried out on pathogenic microorganisms with suspicion of belonging to species that have displayed resistance to commonly used antimicrobial agents. These tests are also very useful for the surveillance of resistance, epidemiology of susceptibility, and comparing new and existing antimicrobial agents. These parameters are very vital in clinical practice in classifying the tested microorganisms as clinically susceptible, intermediate, or resistant to the test antimicrobial agents [12].

Different standard methods have been used to evaluate the antimicrobial activities of plant's crude extracts. However, dilution methods have been favoured over others for the determination of MIC (broth and agar dilutions) and MBC (broth dilution) [12]. Recently, a new method using the oxidation-reduction colourimetric indicator resazurin has been proposed for the determination of drug resistance and MIC of antimicrobial agents against pathogenic organisms [14, 15]. Resazurin, which is blue in its oxidised state, turns pink when reduced by viable cells (Figure 1) and can easily be detected with the naked eyes and the MIC determined even without the aid of a spectrophotometer. This work explores the use of resazurin for the broth microdilution method for the antibacterial MIC determination.

### 4.1. Antibacterial Assay

The agar and broth dilution techniques used in this study revealed that they are both effective antibacterial techniques for the determination of MIC. However, the resazurin broth microdilution technique proved to be more sensitive as it could detect the slightest activity of the organisms where the agar dilution could not; this was evidently observed in the aqueous extract* (V. cholera, S. typhi, *and* E. coli)* in the antibacterial assay (Table 1 and Figure 1).

According to early works on parasitic plants, Hawksworth and Wiens [16] reported that parasitic plants scarcely utilise their photosynthate and depend mostly on the nutrient absorbed from the host. Hence, the biocompounds are chiefly dependent on their host. This was evident in the antimicrobial works of Ogunmefun et al. [5], Deeni and Sadiq [17], and Efuntoye et al. [18] which showed different antimicrobial activities of the same plant on different hosts. This study in comparison to the works of Ogunmefun et al. [5] showed that* Phragmanthera capitata *of rubber tree* (Hevea brasiliensis)* showed greater promise for its antibacterial potential (MIC of 1.25 mg/mL to 5 mg/mL in the organic solvents) than the ones harvested from kola nut and cocoa (MIC of 100 mg/mL to 200 mg/mL). While* Bacillus *sp.*, K. pneumonia, *and* E. coli *were resistant in* P. capitata *from kola nut and cocoa [5], these bacteria were highly susceptible to the same species collected from rubber trees in this work.

The bactericidal ability of* P. capitata* parasitic on rubber tree was also assayed and the result revealed that this plant not only will inhibit these bacteria but also has the potential to kill them at appreciable concentrations (Table 2). Hence,* P. capitata* parasitic on rubber tree has potential as an antibiotic in the pharmaceutical industry and can serve as an alternative remedy to the diseases caused by these bacteria.

In evaluating the extracts effect on the bacteria in the agar dilution method, incubation was further prolonged for 3 days and the results obtained were in comparison with the MBC. Some organisms that were initially inhibited by the extracts after 20 hrs however recovered and continued growth (Table 2). This could be as a result of the antibacterial biocompounds in the extract(s) becoming weak and less active and therefore bacteria that were still alive recovered and continued to grow. This observed outcome could give some insights into the shelf life or duration of action of the extracts against microorganisms. This simply explains that where organisms continued growth, the extracts at those concentrations were not bactericidal to them. Alternatively, organisms that continued growth may not have been properly dissolved in the agar and hence when the activity of the extract reduced beyond their threshold, they regrew. This could be a major advantage of the broth over the agar dilution method as the organism is highly soluble or mixes very well with the broth as they are both liquid.

### 4.2. Antifungal Activity

The activity of the crude extracts of* P. capitata* was also tested against pathogenic fungi using the agar dilution technique. Four pathogenic fungi were tested but only two, namely,* T. tonsurans *and* A. niger*, were susceptible to the organic extracts. While* A. niger* was susceptible in this work, the same was resistant in the reports of Ogunmefun et al. [5] suggesting that* P. capitata* from rubber tree may be more potent than the ones collected from kola nut and cocoa as an antifungal agent. The result on* C. albicans* from this work is in support of Ogunmefun et al. [5] which did not show any activity on the organic solvents used. Interestingly, while the aqueous extract seldom showed antibacterial activity, the same had a very high antifungal activity as it inhibited all four tested fungi (Table 3 and Figure 2) (H4). This could suggest that water was able to extract the antifungal agent in the plant compared with the organic solvent extracts. There were no marked changes observed after prolonging incubation of the plates for 5 days and where changes occurred, the same reason as above could justify it.

## 5. Conclusion

The outcome of this work clearly revealed that* P. capitata* harvested from rubber trees has the ability to inhibit both gram-positive and gram-negative bacteria effectively and also exhibited antifungal ability and is with great promise for use as an antimicrobial agent in folklore. While it was recorded that water was a weak antibacterial extractant in this study for* P. capitata*, contrarily, water would be recommended if the target was fungi. This work further supported the claimed ethnopharmacological uses of* P. capitata* against gastrointestinal infections and other opportunistic diseases of man and animals. Also, worth noting of the potential of* P. capitata* is that it could serve as an alternative remedy in therapeutics as most of the organisms used for this work have had some reports of resistance to conventional drugs. Further studies of* P. capitata *on different host species, in vivo, and possible isolation of biocompounds are recommended to further validate its folkloric potentials.

## Figures and Tables

**Table 1:** Minimum Inhibitory Concentrations (MIC) of the different solvent extracts of *P. capitata* on selected gram-negative and gram-positive bacteria using the agar and resazurin broth dilution assays.

	Agar dilution (16–20 hrs)					Resazurin broth microdilution				
	Act	Met	Eth	Aqu	Cip	Act	Met	Eth	Aqu	Cip
	mg/mL				*µ*g/mL	mg/mL				*µ*g/mL
*Enterococcus faecalis* (+ve)	2.5	2.5	5	>5	≤2	2.5	2.5	5	>10	0.25
*Staphylococcus aureus* (+ve)	5	5	5	>5	≤2	5	5	5	>10	0.0625
*Bacillus subtilis *(+ve)	5	5	5	>5	≤2	2.5	2.5	2.5	>10	0.0625
*Bacillus cereus *(+ve)	2.5	2.5	2.5	>5	≤2	2.5	2.5	5	>10	0.125
*Streptococcus pyogenes *(+ve)	5	5	5	>5	≤2	5	5	5	>10	0.0625
*Vibrio cholera *(−ve)	2.5	2.5	2.5	5	≤2	2.5	2.5	5	>10	0.0625
*Klebsiella pneumonia *(−ve)	2.5	5	5	>5	≤2	2.5	2.5	2.5	>10	0.25
*Pseudomonas aeruginosa *(−ve)	2.5	2.5	2.5	>5	≤2	2.5	2.5	2.5	>10	0.25
*Salmonella typhi *(−ve)	2.5	5	1.25	5	≤2	5	5	5	>10	0.0625
*Escherichia coli *(−ve)	2.5	2.5	1.25	5	≤2	2.5	1.25	2.5	>10	0.0625

Annotations: Act (acetone extract), Met (methanol extract), Eth (ethanol extract), Aqu (aqueous extract), Cip (ciprofloxacin), “>” (value greater than the highest concentration tested), and “≤” (value lesser than or equal to the lowest concentration tested).

**Table 2:** Minimum Bactericidal Concentrations (MBC) and MIC of the agar dilution after 3 days of incubation of the different solvent extracts of *P. capitata* on selected gram-negative and gram-positive bacteria.

	MBC					Agar dilution (after 3 days)				
	Act	Met	Eth	Aqu	Cip	Act	Met	Eth	Aqu	Cip
	mg/mL				*µ*g/mL	mg/mL				*µ*g/mL
*Enterococcus faecalis* (+ve)	5	5	5	>10	>2	5	5	5	>5	4
*Staphylococcus aureus* (+ve)	5	10	10	>10	0.0625	5	>5	>5	>5	≤2
*Bacillus subtilis *(+ve)	5	10	10	>10	0.125	5	>5	>5	>5	≤2
*Bacillus cereus *(+ve)	5	5	10	>10	>2	5	5	>5	>5	≤2
*Streptococcus pyogenes* (+ve)	5	10	10	>10	0.0625	5	>5	>5	>5	≤2
*Vibrio cholera *(−ve)	5	5	5	>10	0.0625	5	5	5	>5	4
*Klebsiella pneumonia *(−ve)	5	5	5	>10	1	5	5	5	>5	4
*Pseudomonas aeruginosa *(−ve)	10	10	10	>10	0.25	>5	>5	>5	>5	≤2
*Salmonella typhi *(−ve)	5	10	10	>10	0.0625	5	>5	>5	>5	≤2
*Escherichia coli *(−ve)	2.5	2.5	2.5	>10	0.0625	2.5	2.5	2.5	>5	≤2

Annotations: Act (acetone extract), Met (methanol extract), Eth (ethanol extract), Aqu (aqueous extract), Cip (ciprofloxacin), “>” (value greater than the highest concentration tested), and “≤” (value less than or equal to the lowest concentration tested).

**Table 3:** Minimum Inhibitory Concentrations (MIC) of the different solvent extracts of *P. capitata* on selected *human* pathogenic fungi.

	2-3 days after incubation					5 days after incubation				
	Act	Met	Eth	Aqu	Nys	Act	Met	Eth	Aqu	Nys
	mg/mL				*µ*g/mL	mg/mL				*µ*g/mL
*Trichophyton mucoides*	>10	>10	>10	1.25	4	>10	>10	>10	1.25	4
*Trichophyton tonsurans*	1.25	10	5	≤0.3125	4	1.25	10	10	≤0.3125	4
*Candida albicans*	>10	>10	> 10	≤0.3125	4	>10	>10	>10	≤0.3125	>16
*Aspergillus niger*	5	10	5	≤0.3125	8	5	10	10	≤0.3125	8

Annotations: Act (acetone extract), Met (methanol extract), Eth (ethanol extract), Aqu (aqueous extract), Nys (nystatin), “>” (value greater than the highest concentration tested), and “≤” (value less than or equal to the lowest concentration tested).
